# Supplementary material for: Erythrocyte sedimentation rate and hemoglobin-binding protein in free-living box turtles (Terrapene spp.)
Source: PLoS One. 2020 Jun 17;15(6):e0234805. doi: 10.1371/journal.pone.0234805 (PMC7299368; doi:10.1371/journal.pone.0234805)
Supplement: S1 Table — N = sample size, K = number of parameters estimated for each model, AICc = Akaike’s information criterion corrected for sample size, ΔAICc = Difference in Akaike’s information criterion compared to the most parsimonious model, wi = Akaike weight. (DOCX) [file pone.0234805.s002.docx]

**Table S1**. Model selection parameters for general linear models predicting erythrocyte sedimentation rate in free-living eastern box turtles (*Terrapene carolina carolina*). N = sample size, K = number of parameters estimated for each model, AIC_c_ = Akaike’s information criterion corrected for sample size, ΔAIC_c_ = Difference in Akaike’s information criterion compared to the most parsimonious model, w_i_ = Akaike weight.

| **Model** | **N** | **K** | **AIC_c_** | **ΔAIC_c_** | **w_i_** |
| --- | --- | --- | --- | --- | --- |
| **Winpette** |  |  |  |  |  |
| Sex + Season + Packed Cell Volume + Physical Exam | 90 | 6 | 240.33 | 0 | 1 |
| Sex + Season + Packed Cell Volume | 90 | 5 | 253.11 | 12.78 | 0 |
| Physical Exam | 90 | 3 | 259.19 | 18.86 | 0 |
| Sex + Physical Exam | 90 | 4 | 259.49 | 19.17 | 0 |
| Null | 90 | 2 | 275.62 | 35.29 | 0 |
| Sex | 90 | 3 | 276.36 | 36.03 | 0 |
| **Winpette Calipers** |  |  |  |  |  |
| Sex + Season + Packed Cell Volume + Physical Exam | 90 | 6 | 228.16 | 0 | 1 |
| Sex + Season + Packed Cell Volume | 90 | 5 | 240.26 | 12.1 | 0 |
| Physical Exam | 90 | 3 | 260.02 | 31.86 | 0 |
| Sex + Physical Exam | 90 | 4 | 260.28 | 32.12 | 0 |
| Null | 90 | 2 | 275.42 | 47.26 | 0 |
| Sex | 90 | 3 | 276.1 | 47.94 | 0 |
| **Microhematocrit Tube** |  |  |  |  |  |
| Sex + Season + Packed Cell Volume + Physical Exam | 89 | 6 | 302.63 | 0 | 0.99 |
| Sex + Season + Packed Cell Volume | 89 | 5 | 312.36 | 9.73 | 0.01 |
| Sex + Physical Exam | 89 | 4 | 318.66 | 16.03 | 0 |
| Physical Exam | 89 | 3 | 319.84 | 17.2 | 0 |
| Sex | 89 | 3 | 332.5 | 29.86 | 0 |
| Null | 89 | 2 | 332.99 | 30.36 | 0 |
