# Supplementary material for: Erythrocyte sedimentation rate and hemoglobin-binding protein in free-living box turtles (Terrapene spp.)
Source: PLoS One. 2020 Jun 17;15(6):e0234805. doi: 10.1371/journal.pone.0234805 (PMC7299368; doi:10.1371/journal.pone.0234805)
Supplement: S2 Table — N = sample size, K = number of parameters estimated for each model, AICc = Akaike’s information criterion corrected for sample size, ΔAICc = Difference in Akaike’s information criterion compared to the most parsimonious model, wi = Akaike weight. (DOCX) [file pone.0234805.s003.docx]

**Table S2.** Model selection parameters for general linear models predicting erythrocyte sedimentation rate in free-living ornate box turtles (*Terrapene ornata ornata*). N = sample size, K = number of parameters estimated for each model, AIC_c_ = Akaike’s information criterion corrected for sample size, ΔAIC_c_ = Difference in Akaike’s information criterion compared to the most parsimonious model, w_i_ = Akaike weight.

| **Model** | **N** | **K** | **AIC_c_** | **ΔAIC_c_** | **w_i_** |
| --- | --- | --- | --- | --- | --- |
| **Winpette** |  |  |  |  |  |
| Sex + Packed Cell Volume + Physical Exam | 104 | 5 | 293.46 | 0 | 0.91 |
| Sex + PCV | 104 | 4 | 298.61 | 5.15 | 0.07 |
| Sex | 104 | 3 | 301.38 | 7.92 | 0.02 |
| PE | 104 | 3 | 305.91 | 12.45 | 0 |
| Null | 104 | 2 | 309.56 | 16.1 | 0 |
| **Winpette Calipers** |  |  |  |  |  |
| Sex + Packed Cell Volume + Physical Exam | 85 | 5 | 193.15 | 0 | 0.94 |
| Sex + Packed Cell Volume | 85 | 4 | 199.23 | 6.08 | 0.04 |
| Physical Exam | 85 | 3 | 203.23 | 10.08 | 0.01 |
| Sex | 85 | 3 | 203.51 | 10.37 | 0.01 |
| Null | 85 | 2 | 207.25 | 14.1 | 0 |
| **Microhematocrit Tube** | |  |  |  |  |
| Sex + Packed Cell Volume + Physical Exam | 104 | 5 | 288.97 | 0 | 0.97 |
| Sex + Packed Cell Volume | 104 | 4 | 295.97 | 7 | 0.03 |
| Sex | 104 | 3 | 305.12 | 16.15 | 0 |
| Physical Exam | 104 | 3 | 307.05 | 18.08 | 0 |
| Null | 104 | 2 | 311.28 | 22.31 | 0 |
