# Supplementary figures and images for: Erythrocyte sedimentation rate and hemoglobin-binding protein in free-living box turtles (Terrapene spp.)
Source: PLoS One. 2020 Jun 17;15(6):e0234805. doi: 10.1371/journal.pone.0234805 (PMC7299368; doi:10.1371/journal.pone.0234805)

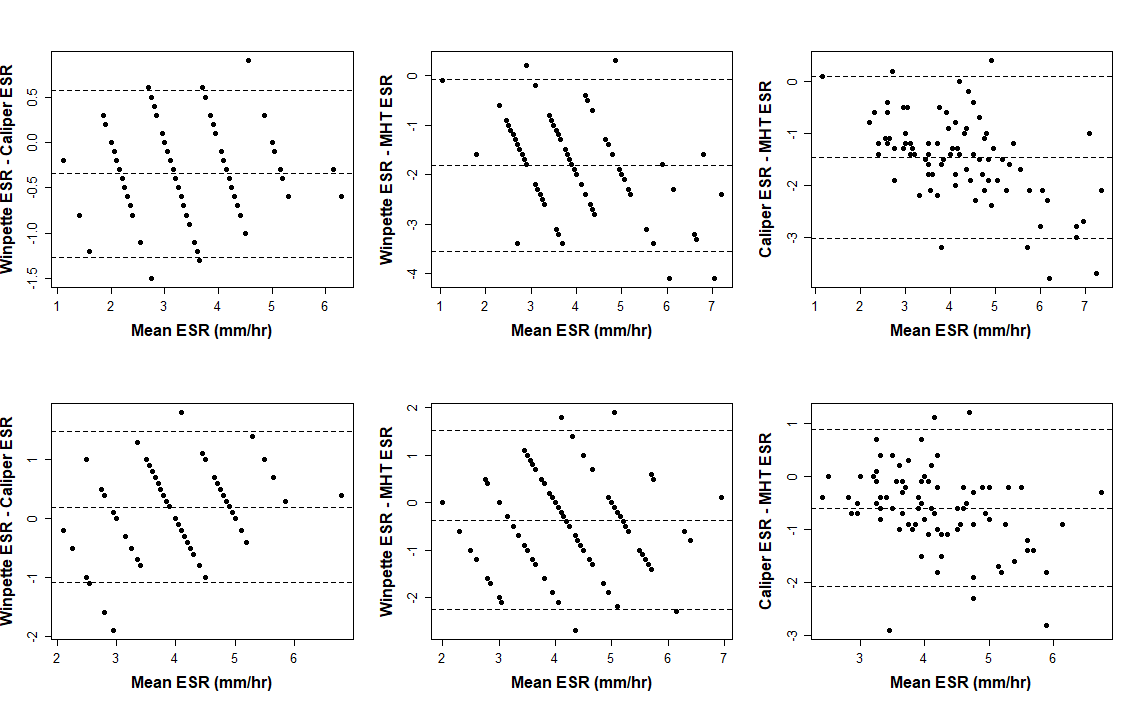

Supplement: S1 Fig — Top row: T. carolina, Bottom row: T. ornata. Central dashed line = Mean difference between measurement methodologies, Top and bottom dashed lines = limits of agreement, defined as the mean difference +/- 1.96 times the standard deviation of the differences. MHT = microhematocrit tube ESR. (TIF) [file pone.0234805.s004.tif]

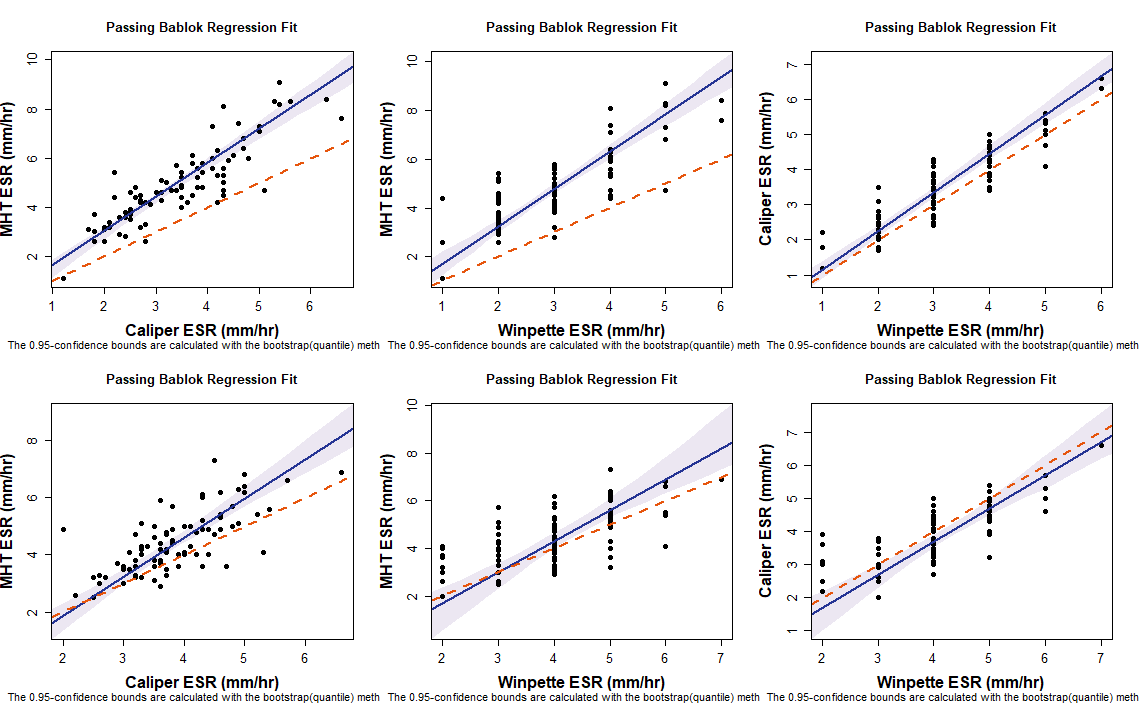

Supplement: S2 Fig — Top row: T. carolina, Bottom row: T. ornata. Dashed line = line of perfect agreement with slope = 1 and y-intercept = 0. Solid line: Passing-Bablok regression line. Shaded region: 95% confidence interval of Passing-Bablok regression line. MHT = microhematocrit tube ESR. (TIF) [file pone.0234805.s005.tif]
