# Supplementary material for: Erythrocyte sedimentation rate and hemoglobin-binding protein in free-living box turtles (Terrapene spp.)
Source: PLoS One. 2020 Jun 17;15(6):e0234805. doi: 10.1371/journal.pone.0234805 (PMC7299368; doi:10.1371/journal.pone.0234805)
Supplement: Attachment — Submitted filename: Adamovicz_box_turtle_ESR_HBP_rebuttal.docx [file pone.0234805.s006.docx]

Dr. Munderloh and anonymous peer-reviewers,

Thank you for your thoughtful reviews of our manuscript. We have addressed each of your comments and feel that the paper is overall improved. Please see our responses to specific points below. We look forward to your thoughts on the new draft!

Sincerely,

Laura Adamovicz, DVM, PhD

Research Scientist, Veterinary Diagnostic Laboratory

Wildlife Epidemiology Section

University of Illinois

2001 S. Lincoln Ave

Urbana, IL 61802

**Reviewer #1:**

**Title: Many of these are not new diagnostics, they a quite old, just new for this species. I addition as written the title is overly vague. Suggested title revision: Comparison Acute Phase Reactants for Health Assessment of free-living box turtles (Terrapene spp,)**

We take the reviewer’s point on specificity of language, but disagree that the proposed title is an improvement, as the purpose of the paper was not to compare ESR to HBP. Please see the revised, unambiguous title on lines 1-2.

**Throughout the manuscript, sentences should not begin with acronyms. I count 13 uses of acronyms in the abstract; reduce the use of acronyms in the abstract to only those absolutely necessary. I recommend removal of APR, OBT/EBT (use shortened scientific name instead) and using acronyms only for tests to increased abstract readability.**

Changed as requested

**Line: 209, 239, 344, 370, 123, 268, 279, 331382,
236 provides an example of oververbosity which can and should be summarized throughout the MS. Please review the entire text for these instances: Results for the single juvenile EBT were 4, 4.1, and 6 mm/hr for the Winpette, caliper measured Winpette and the Microhematocrit methods, respectively.**

Language in the sentences at lines 209 (now 224), 239 (now 253), 279 (now 290), and 344 (now 439) has been revised and shortened as requested, however, some of these sentences are already quite short and were not changed. Examples:

Line 123 “Erythrocyte sedimentation rate was performed with a commercially available kit using the Wintrobe method (Winpette, Guest Scientific AG, Switzerland).”

Line 268: “Hemoglobin-binding protein was assayed in 184 OBT from one study site over the course of three years (Table 1, Fig 2).”

Line 331: “Hemoglobin-binding protein is higher in male ringed seals and rhesus macaques (Macaca mulatta) than females, while the reverse is true for T. carolina [23,76,78].”

Line 382: “Hemoglobin-binding protein was affected by health status and age class.”

If this list of line numbers was actually a list of sentences starting with acronyms, those have also been revised in the updated draft.

The text at lines 236 – 239 (now 250 – 253) has been revised.

**Line 102 - provide field sites in additional files**

We prefer for these sites to remain out of the official publication record. If someone needs this information for a valid reason, they can contact the corresponding author to get it. There has been evidence of turtle poachers using the scientific literature to identify prime spots for animal acquisition, and we do not wish to contribute to this problem.

**Line 109 without clinical signs**

This sentence is describing how turtles were classified as “unhealthy”, and the correct phrasing is “with clinical signs”. To make this more obvious, the wording was changed to “displaying clinical signs”.

**Line 115 - provide full details was pain control provided**

Shell notching is a straightforward and commonly-used procedure for permanent identification of turtles which is approved by the University of Illinois IACUC. Shell notching consists of cutting a small notch or series of notches (not exceeding 4 per individual) in the marginal scutes using a hand-held hacksaw blade which is disinfected between turtles using 2% chlorhexidine. The notches are no larger than 3mm x 3mm, and each notch takes only a few seconds to make. Pain associated with this procedure is generally assumed to be temporary and minimal, and analgesics are not routinely administered by us or others who report the use of this technique in the literature. Nociception is poorly understood in reptiles, and data on efficacy, safety, and dosage for analgesic drugs are scant. Documented side effects (e.g. respiratory depression with opioid administration) which are minimally concerning for chelonians in a clinical setting could be significant and potentially life-threatening for wild animals with exposure to environmental hazards and predators. Furthermore, relay toxicities have been documented for scavengers consuming animals treated with analgesics before death (e.g. *Gyps* vultures consuming diclofenac-treated cattle), and the environmental safety of many drugs used to treat reptiles is uncharacterized. Because we release turtles immediately after processing and we are unable to monitor their condition following release, analgesics are not administered. Shell notches do not appear to affect behavior, generally heal within a few weeks, and complication rates (i.e. localized infection) are very low.

We have added some additional detail to the text as requested at lines 118 – 121.

**Line 113-115 - which test was determined from which, separator gels can interfere with some assays**

The requested information has been added (now lines 115 – 118). While the reviewer is correct about potential assay interference due to separator gels in certain situations, the PHASE haptoglobin kit instructions specifically recommend using lithium heparin plasma separator tubes. We therefore consider it unlikely that the gel causes any significant interference with this test.

**Line 128 -Provide full details, make manufacturer**

Details added as requested.

**Line 141 - The differences in time of sample storage and results should be assessed statistically**

Results of a general linear model with HBP concentration as the dependent variable and storage time as the independent variable are now reported at lines 293 – 294. HBP concentration was not significantly associated with storage time.

**Line 134- provide full details, make manufacture of "stand"**

Information added as requested.

**Line 167 - provide full details, AICmodavg package**

Reference 72 (at the end of the referenced sentence) contains the full details for this R package.

**Line 177 - ReferenceIntervals package: provide full details**

Added as requested

**Line 346 - You have not proven this for box turtles**

This statement has been removed

**Line 292- 294 Inter-assay variation is generally provided as the beginning of the results and reviewed justified in the discussion as acceptable or unacceptable based on previous studies. further discussion appears lacking.**

This section of text has been moved to the beginning of the results section as requested. Additional discussion has been added at lines 322 – 335.

**Line 355 - you didn't vary your anticoagulant in this study...**

This sentence has been rephrased to the following: “Erythrocyte sedimentation rate values can vary by both anticoagulant and measurement method.”

**In the goals of the study and with the discussion you state you " Characterized the spatial, temporal, demographic, and physiologic factors which influenced test interpretation". this statement both within goals and discussion is overly broad, you characterized selected, or some factors: In the discussion and the introduction I would specify which you factors tested and which category you believe they belong to.**

Please see revised text in the introduction (lines 91-92) and discussion (lines 316-319).

**Additional points for the discussion: Justify your choice of the SCC considering: Jugular vein is known to have less lymphatic contamination and no CSF contamination. Justify determination method of determination of lack of lymphatic contamination or other ways this could have been determined. Was hemolysis or lipemia assessed in the study and if so or if not how might this have affected results, shouldbe in discussion**

The reviewer is correct that the risk of sample contamination with other fluids is lower (but not non-existent) when blood is collected from the jugular vein. We chose to use the subcarapacial sinus for a few reasons. First and foremost, this sampling site is readily accessible even in box turtles that are fully closed. Accessing the jugular vein requires opening the shell, fully extending the head, and restraining the forelimbs. This is time consuming, more stressful for the animal, and poses a greater risk of injury to both the turtle and the handlers. Furthermore, jugular veinpuncture requires two people – one to restrain the turtle and one to collect the blood sample. Drawing from the subcarapacial sinus can be accomplished by a single person. Finally, jugular venipuncture is more technically challenging than blood collection from the subcarapacial sinus. The jugular vein is an isolated vessel that is frequently not visible, requiring a “blind stick”. In contrast, the subcarapacial sinus is a confluence of vessels (thus a larger target) with clear anatomic landmarks. While sampling from the jugular vein would be most ideal, we sometimes process up to sixty turtles in a day, and this makes jugular venipuncture impractical. The subcarapacial sinus is a reasonable alternative that meets our methodological needs, with the admitted limitation of potential sample contamination. Please see added text at lines 467 - 474.

Blood samples that were visually contaminated with lymphatic fluid were identified during venipuncture and excluded from analysis. Visualization of clear or yellow-tinged fluid entering the syringe immediately before or along with blood is the best way to identify lymphatic contamination, though as we state in the text, lower levels of lymph contamination could go undetected using this method alone. Decreases in packed cell volume with no evidence of a regenerative erythroid response and increases in lymphocytes can be appreciated in lymph-diluted sampled on hematology. Biochemistry panel differences are also variably reported for lymph-contaminated samples. While we could have pursued additional diagnostics to support our visual assessment of lymphatic contamination, there is no way to ever fully rule it out. Additional diagnostics were beyond the scope of this study, and as we state in the discussion, the possibility of lymphatic contamination is a recognized limitation in this manuscript. Please see added text at lines 474 - 479.

Samples were visually examined for lipemia (though this is rare in reptiles) and hemolysis by noting the color of the plasma in the PCV tubes, Winpettes, and plasma separator tubes. Samples with visible hemolysis or lipemia were excluded from the study. We have added a description of these methods at lines 163 - 164.

**Line 120: it appears that samples were moved from a heparinized tube into yet another heparinized to for HCT determination, increasing the likelihood of hemolysis, was this assessed?**

Though not explicitly stated in the original manuscript, sample quality was evaluated at every step of laboratory analysis and samples displaying any evidence of alteration (contamination with CSF or lymphatic fluid, clotting, hemolysis, lipemia) were excluded from analysis. PCV was determined using heparinized capillary tubes, and no hemolysis was noted. The placement of heparinized box turtle blood into heparinized capillary tubes for PCV determination has been routinely performed in our lab for hundreds of animals, and hemolysis is uncommon in our experience.

**311: You suggest this may be due to erythrocyte frictional forces as in other species but do not know this to be true in these current study. Other factors which also change based on sex a reproduction could also change ESR: increased proteins, lipoproteins and immunoglobulins of vittellogensis could affect ESR.**

The sentence beginning at line 311 (now 340) refers to the general relationship between PCV and ESR, while sex-based findings are discussed in subsequent sentences. We have weakened the language in sentence 340 to reflect uncertainty in the mechanism producing this finding. We have also added a sentence at lines 348 – 349 postulating additional potential causes for the relationship between ESR and sex in ornate box turtles.

**Figs: ESR and Haptoglobin - Provide scientific names as well as common names. Consider additional figures of ectotherms only (or reptiles only, preferred) with box plot to better detail difference among species more closely taxonomically related. Some reference are cited within the figure, others are not. I suggest provide all reference citation at the end of the Figure titles for these comparative figures.**

The scientific names have been added as requested. They were originally not included for simplicity and ease of readability, but we will defer to the editor’s decision on which versions are preferred for final publication. Box plots would require either access to the original data from multiple publications, or availability of the minimum, maximum, and quartile values for each dataset. This information was not consistently provided by the cited references, so we elected to use measures of central tendency and dispersion instead, as those were available in every study. We agree that it would be nice to have more detailed information comparing reptilian values, but it is just not available in every instance. Thank you for catching the remaining superscripted numbers in Figure 2 – that was an editing error on our part. All values are referenced in the figure titles.

**Tables have excessive acronyms. EBT, OBT within the table should be replaced with the scientific name OR the common name with the scientific name give in the Table info**

Changed as requested.

**Table 2 NEW TITLE: Effect of Free-Living Box Turtle Sex, Physical Examination, and Packed Cell Volume upon Erythrocyte Sedimentation Rates based on multivariable general linear models. Winpette, Winnpette C and ( MicroHCT should be expanded within the table. Se, T, The test for the P value and Effect size should be defined.** **Are the miniscule changes found to be statistically different here really clinically revelant, what constitutes clinical relevancy in other species for these tests? This should be addressed in the discussion**

The table title has been revised. We have added text to define the effect size, which is a marginal mean estimate from the most parsimonious multivariable general linear model for each species (Tables S1 & S2). Abbreviations have been expanded as requested.

The reviewer is correct to point out that statistical significance does not always signify biological importance. However, in this instance we feel that our findings may have clinical relevance for a few reasons. First, “unhealthy” turtles had more than one reason for presentation – while trauma was most common, other turtles had clinical signs consistent with microbial or viral infection (granulomas, oral ulcers, peeling and discolored scutes). This variability in presenting complaint may have influenced the degree of ESR and HBP elevation. Second, “unhealthy” turtles were sampled at different stages and severities of illness or injury, potentially diluting the magnitude of ESR and HBP deviations. The fact that this heterogeneous “unhealthy” group still had higher ESR and HBP values than their apparently healthy counterparts demonstrates the potential clinical utility of these tests. This is especially true because other reptilian diagnostics do not consistently differentiate between healthy and unhealthy states, as described in the introduction. While the sample size for “unhealthy” turtles in this study was too small to draw firm conclusions about clinical relevance, our findings have prompted follow-up studies to evaluate ESR and HBP in large numbers of sick and injured turtles. These studies will provide valuable information about the diagnostic performance, prognostic capabilities, and clinical utility of each test.

Please see the added text at lines 360 – 374 and 392 – 438 which compare the effect sizes in this study to those in the existing literature and provide additional discussion about the potential clinical relevance of our findings.

**Table 3: You have plant of space within the table to spell out and provide the scintific name of each species
A less shortened form of each method could also be used within the table. Last Column Header Proportional error, results Yes or No below.**

Abbreviations have been removed as requested. Instead of changing the column heading, we have spelled out “proportional” within the table.

**Table 4 : Title should be revised into summary form, there is much redundancy. Method and Age should be different columns. Suggest separate tables for HBP and ESR as there is no benefit in placing them together.**

Tables have been separated and changes have been made as requested.

**ESR is measured as a rate and is therefore either faster or slower than OR relatively increased or decreased in rate compared to other measurements. It is not higher (elevated) or lower. Please correct throughout the manuscript.**

According to the International Council for Standardization in Haematology and the Clinical and Laboratory Standards Institute ESR values should be reported as millimeter measurements at one hour, instead of mm/hr. I have corrected this throughout the draft. Since ESR values are reported as distances, not rates, we have referred to them using “greater / shorter” terminology throughout the draft.

**HBP is measured as a concentration and is therefore has a relatively increased or decreased concentration in comparison to other concentrations; it is not higher (elevated) or lower. Please correct throughout the manuscript.**

Changed as requested.

**Tables S1 & S2: Model attributes are not defined**

Added as requested.

**Reviewer #2:**

**Line 94-95: I'm not sure for the rationale behind expectations and predictions, especially that of females. Please describe more context behind the specific hypotheses in this study, so that this study reads as more than simply being a technological question of comparing multiple assays in free-living turtles.**

References have been added for each hypothesis, and each is reviewed in detail within the discussion section.

**Linen 284: “data” is a plural word, thus this should state “summary data are”**

Corrected as requested
